# Expression of Epidermal Growth Factor Receptor Detected by Cetuximab Indicates Its Efficacy to Inhibit *In Vitro* and *In Vivo* Proliferation of Colorectal Cancer Cells

**DOI:** 10.1371/journal.pone.0066302

**Published:** 2013-06-18

**Authors:** Kohei Shigeta, Tetsu Hayashida, Yoshinori Hoshino, Koji Okabayashi, Takashi Endo, Yoshiyuki Ishii, Hirotoshi Hasegawa, Yuko Kitagawa

**Affiliations:** Keio University, School of Medicine, Department of Surgery, Shinjuku-ku, Tokyo, Japan; The Chinese University of Hong Kong, Hong Kong

## Abstract

Cetuximab is a chimeric mouse–human monoclonal antibody that targets the human epidermal growth factor receptor (EGFR). However, EGFR expression determined by immunohistochemistry does not predict clinical outcomes of colorectal cancer (CRC) patients treated with cetuximab. Therefore, we evaluated the correlation between EGFR levels detected by cetuximab and drug sensitivities of CRC cell lines (Caco-2, WiDR, SW480, and HCT116) and the A431 epidermoid carcinoma cell line. We used flow cytometry (FCM) to detect EGFR-binding of biotinylated cetuximab on the cell surface. Subcloned cell lines showing the highest and lowest EGFR expression levels were chosen for further study. Cytotoxic assays were used to determine differential responses to cetuximab. Xenograft models treated with cetuximab intraperitoneally to assess sensitivity to cetuximab. Strong responses to cetuximab were specifically exhibited by subcloned cells with high EGFR expression levels. Furthermore, cetuximab inhibited the growth of tumors in xenograft models with high or low EGFR expression levels by 35% and 10%–20%, respectively. We conclude that detection of EGFR expression by cetuximab promises to provide a novel, sensitive, and specific method for predicting the sensitivity of CRC to cetuximab.

## Introduction

The epidermal growth factor receptor (EGFR) is a member of the human EGFR family of receptor protein tyrosine kinases. It is an important therapeutic target in metastatic colorectal cancer (mCRC), and increased EGFR expression is the hallmark of many human tumors [1], [2]. Activation of the EGFR signaling pathway results in increased tumor proliferation, angiogenesis, metastasis, and tumor invasiveness through the binding of a number of different ligands, including EGF-like molecules, transforming growth factor-α (TGFα), and neuregulins to the receptor’s ectodomain [3]. EGFR activation results in the initiation of potentially oncogenic intracellular signaling cascades, including the RAS-mitogen-activated protein kinase (MAPK), phosphoinositide 3-kinase (PI3K)/Akt, phospholipase C, signal transducer and activator of transcription (STAT), and SRC/FAK pathways [4]–[6].

The development of monoclonal antibodies has improved the strategies for inhibiting the activity of the EGFR inhibition in cancer therapy. Cetuximab (Erbitux, Merck-Serono, Darmstadt, Germany) is a chimeric monoclonal antibody (IgG1) that binds to the ectodomain of the human EGFR and competitively inhibits ligand binding to suppress tumor proliferation. The efficacy of cetuximab was evaluated in combination with irinotecan to treat mCRC patients whose tumors are positive for EGFR expression (assessed by immunohistochemistry) and are resistant to FOLFOX or FOLFIRI regimens [7]–[10].

Many studies have been conducted to identify factors that can predict the response to treatment, and CRC with mutated *KRAS* was identified as a rule does not respond to anti-EGFR therapy. [11], [12]. In contrast, factors such as EGFR over-expression, amplification of its gene, and p53 mutations correlate with the response to cetuximab; however, they are not completely effective in predicting the response to cetuximab therapy [13], [14]. Experimental studies have suggested a correlation between the EGFR expression level and the efficacy of cetuximab [15]. However, this correlation seems to be speculative in the clinical setting, and some studies report that no relationship has been found between the intensity of the immunohistochemical staining for EGFR and the response rate [assessment was performed using Response Evaluation Criteria in Solid Tumors (RECIST)], progressive free survival, or overall survival in clinical trials [8], [16]–[18].

A recent report demonstrated that a mutation within the EGFR ectodomain confers resistance to cetuximab by preventing its binding [19]. Therefore, we speculated that the detection of EGFR using immunohistochemical staining using non-specific IgG1 antibody differs from detection by cetuximab. We further hypothesized that the cell membrane-specific EGFR expression levels, which can be detected by cetuximab, may influence the inhibition of cell proliferation. In this study, we devised a method, in which we used biotinylated cetuximab as the primary antibody for flow cytometry (FCM) to directly detect the EGFR expression by CRC cell lines. Using this technique we evaluated the relationship between EGFR levels detected by cetuximab-sensitivities of CRC cell lines.

## Materials and Methods

### Preparation of Biotinylated Cetuximab

**Figure 1:**
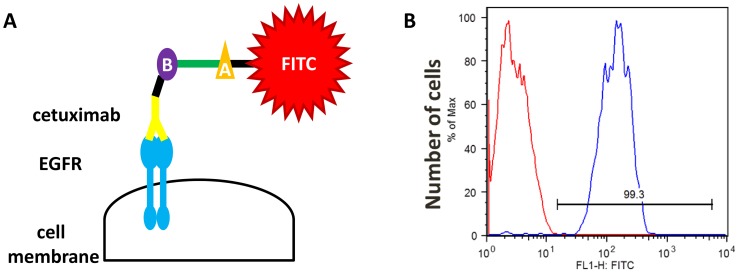
Description and validation of a new method for detecting EGFR expression using biotinylated cetuximab. (A) Biotinylated cetuximab is used as a primary antibody to detect EGFR, and an avidin-FITC secondary antibody is used during FCM to detect antigen–antibody complexes. (B: biotin, A: avidin) (B) FCM analysis of EGFR expression by A431 cells. Controls with antihuman, nonspecific IgG1 antibody labeled with FITC are described in red and cetuximab-biotylated antibody in blue. Fluorescence intensity is approximately 5-times higher relative to the control antibody.

The mechanism by which biotinylated cetuximab binds to EGFR is shown in Figure 1a. Biotin was conjugated to cetuximab using an adaptation of the method described by Medical & Biological Laboratories Co., Ltd.

### Colon Cancer Cell Lines and Identification of EGFR Ectodomain Mutations, *KRAS*, *BRAF*, and *PIK3CA*

Four human CRC cell lines, Caco-2, WiDR, SW480, HCT116, and epidermoid carcinoma cell line A431 were obtained from the American Type Culture Collection (ATCC, Manassas, VA, USA) and cultured as recommended. Authentication of all cell lines was performed by investigating the mutation status of each CRC cell line using the Scorpion-arms or direct sequence methods (conducted by SRS Co., Japan). *KRAS* (codons 12 and 13), *BRAF* (exon 15), PIK3CA (exons 9 and 20), and EGFR ectodomain (S492) mutations were determined in a subset of cell lines and results are summarized in Table 1.

**Table 1:** Mutational status of CRC cell lines.

	Caco-2	WiDR	SW480	HCT116
**KRAS**	Wild-type	Wild-type	Codon G13D	Codon G13D
**BRAF**	Wild-type	Exon 15 (V600E)	Wild-type	Wild-type
**PIK3CA**	Wild-type	Wild-type	Exon 9 (E545K/D)	Exon 20 (H1047R)
**EGFR ectodomain**	Wild-type	Wild-type	Wild-type	Wild-type

Caco-2, WiDR, SW480, and A431 were grown in Dulbecco’s Modified Eagle medium (DMEM) supplemented with 10% fetal bovine serum (FBS) and 5% 0.1 mM penicillin–streptomycin, and HCT116 was grown in Roswell Park Memorial Institute (RPMI) medium supplemented with 10% FBS and 5% 0.1 mM penicillin–streptomycin. All cell lines were incubated at 37°C in a humidified atmosphere containing 5% CO_2_.

### EGFR-binding of Biotinylated Cetuximab and Evaluation of EGFR Expression Levels using FCM

We used biotinylated cetuximab as the primary antibody for FCM. To confirm cetuximab binding to EGFR, FCM was performed using the A431 cell line that expresses high levels of EGFR. A431 cells were adjusted to 1×10^6^ cells/tube and washed twice with 2 mL of ice-cold phosphate-buffered saline (PBS), and blocking was performed for 30 min using Blocking Reagents N102 (NOF Co., Tokyo, Japan). The cells were washed again and then biotinylated cetuximab was added for 1 h to cells that were kept on ice. An antihuman, nonspecific IgG1 antibody labeled with fluorescein isothiocyanate (FITC) was used as control. After washing the cells with ice-cold PBS, an avidin-FITC antibody (Streptavidin, Alexa Fluor® 488 conjugate, Molecular Probes®), a secondary antibody, was added for 15 min on ice. Finally, propidium iodide (PI) was used to determine cell viability.

A BD FACS Aria™ III cell sorter system (BD Biosciences, San Jose, CA, USA), was used for FCM. EGFR expression levels were evaluated by calculating the intensity of the FITC signal. After evaluating the binding of biotinylated cetuximab to A431 cells, EGFR expression levels of the remaining four CRC cell lines were assessed by the same method. Furthermore, cytograms (comparison of FITC and PE signal) and histograms for each CRC cell line were compared to those for A431 cells using a BD FACSDiva 6.0 (BD Biosciences).

### Establishing Subclones with High and Low EGFR Expression Levels

Limiting dilution of cell cultures was conducted in 96-well tissue culture plates seeded at 0.8 cells/well in conditioned media (DMEM supplemented with 10% FBS and 5% 0.1 mM penicillin–streptomycin) harvested from healthy (>1×10^6^ cells/mL and >95% viability) CRC cell cultures. Twenty subclones of each CRC cell lines were isolated, and EGFR expression levels were determined using biotinylated cetuximab as described above. Cells with either the highest or the lowest EGFR expression levels were chosen and cultured for proliferation assays and used in the xenograft model. The mutation status of each subcloned CRC cell lines were investigated again to determine whether there is any difference between wild and subclone cells.

### Growth Suppression Assay

Cells (1,000 and 3,000 cells/well) were seeded immediately after the evaluation of FCM in the wells of a 96-well plate in DMEM or RPMI medium as mentioned above supplemented with 0.5% FBS and 5% penicillin–streptomycin. Cells were treated with various concentrations of cetuximab after 24 h and incubated for 6 days. Cell viability was determined using the 3-(4, 5-dimethylthiazol-2-yl)-2, 5-diphenyltetrazolium bromide assay, and the formation of formazan was measured by its absorbance at 550 nm. The relative rate of cell growth for each cell line was factored into the analysis by subtracting the absorbance at time 0 from those of the control and treatment groups.

### Xenograft Models and Cetuximab Treatment

All animal experiments in this study were approved by the Animal Care and Use Committee of Keio University. Subcloned cell lines derived from WiDR and SW480 (2.0×10^6^ cells) were injected subcutaneously into the right and left sides of the back of 5-week-old female nude mice. The expression level of EGFR of these subcloned cells was assessed by FCM in each time of experiment and the cells were immediately injected. Tumor size was measured using a caliper, and the volume was calculated using the following formula: Volume = length×width×height. Mice were treated with 30 mg/kg cetuximab administered intraperitoneally on days 0, 7, 14, and 21 or with the vehicle control PBS (same quantity as cetuximab). The treatment was initiated when the tumor size was approximately 100 mm^3^. Tumor sizes were measured twice each week until day 28, and the ratio of tumor volumes to those determined on day 0 was calculated.

We evaluated the EGFR expression in the post-treatment tumors by immunohistochemical staining using anti-human, wild-type EGFR antibody and not cetuximab. Processing of the tissue samples was done using tissue processor (Sakura RH-12DM-II, Japan). Briefly the tissue samples were fixed in 10% formalin for 24 h, and then processed in an ascending series of ethanol and subsequently cleared with xylene and embedded in paraffin. The paraffin embedded tissue samples were sectioned at a thickness of 4 µm using a microtome (YAMATO ROM-380, Japan). The sections were mounted on starfrost/silane coated slides (MUTO PURE CHEMICALS NewSilane II, Japan) and air-dried. On the day of staining the slides were immersed in xylene for 10 min before rehydration in ethanol series. Sections were incubated in hydrogen peroxide for 10 min to block endogenous peroxidase activity. Pretreatment of deparaffiinized tissue sections with heat-induced epitope retrieval is required. Optimal results are obtained by pre-treating tissues with with heat-induced epitope retrieval using TE buffer, pH 9.0. After which, the sections were incubated with Anti-Human Wild-Type EGFR (Dako, USA) primary antibody (1∶50) for 4 degree overnight. To confirm the specificity of binding, normal mouse serum IgG was used as negative control instead of primary antibody. Following extensive washing, sections were incubated for 1 hr in the secondary biotinylated antibody followed by DAB Chromogen (Dako REAL EnVision Detection System, USA) for 8 min. Sections were then counter-stained with Mayer’s hematoxylin and dehydrated in ascending grades of ethanol before clearing in xylene and mounting under a cover slip. EGFR cytoplasmic membrane positivity was considered positive EGFR staining. Staining intensity was scored as 0 (no staining), 1+ (weak), 2+ (moderate) and 3+ (strong).

### Statistical Analyses

All statistical analyses were performed using Stata software Version 11.0. Differences between two groups were analyzed using an unpaired Student’s *t* test and *p*<0.05 was considered statistically significant.

## Results

### Detection of EGFR by Biotinylated Cetuximab

The ability of biotinylated cetuximab to detect EGFR using FCM was evaluated using the A431 cell line, which expresses high levels of EGFR [20]. Biotinylated cetuximab was used as the primary antibody and FCM was performed to detect the FITC signal. Strong FITC intensity was detected compared with the control FITC-labeled IgG (Fig. 1B). This finding suggests that EGFR expressed by the A431 cell line can be evaluated using biotinylated cetuximab.

### Mutation Status of *KRAS*, *BRAF*, *PIK3CA*, and EGFR S492 Ectodomain

The mutation status of each CRC cell line is shown in Table 1. *KRAS* mutations in codons 12 and 13, which predict the efficacy of cetuximab treatment, were not detected in Caco-2 and WiDR. In contrast, the codon G13D mutation was detected in SW480 and HCT116. The codon 12 mutation was not detected in the four CRC cell lines. The exon 15 mutation in *BRAF* was only detected in WiDR. Furthermore, *PIK3CA* mutations in exons 9 or 20 were detected in SW480 and HCT116, respectively. Finally, EGFR S492 ectodomain mutation was not detected in all cell lines.

From these results, we chose Caco-2 as the cell line because it has no mutation in any of the three genes and is expected to be sensitive to cetuximab. HCT116 was used as a negative control because it has the PIK3CA mutation in exon 20, which has already been shown to be resistant to cetuximab [21], [22]. On the other hand, SW480 with a KRAS mutation in p.G13D was selected, since it is known that p.G13D-mutated tumors are more sensitive to cetuximab compared with other KRAS-mutated tumors [23]. We had a strong interest in this mutation and wanted to evaluate whether our hypothesis and experimental model could be applied to p.G13D-mutated tumors. Furthermore, we were also interested in the effect of cetuximab with BRAF mutation because sensitivity to cetuximab was previously observed in the xenograft model of BRAF-mutated tumors [24].

### Evaluation of EGFR Expression Levels in CRC Cell Lines

**Figure 2:**
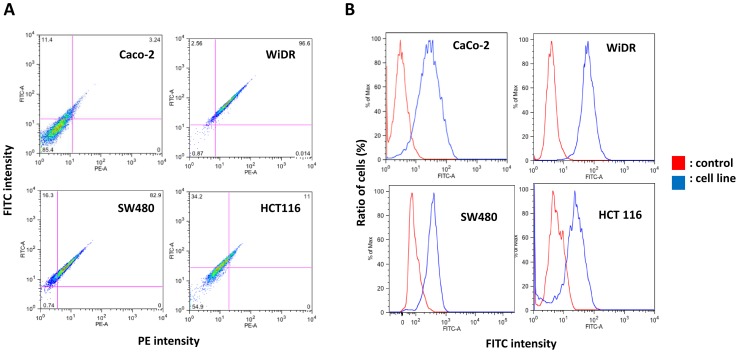
EGFR expression levels in CRC cell lines. (A) Each cell line was evaluated by FCM with biotinylated cetuximab. All four CRC cell lines differed in EGFR expression levels. (B) Histogram of each CRC cell lines. Expression level of EGFR was measured by the intensity of FITC fluorescence.

EGFR expression levels in each CRC cell line were evaluated using FCM with biotinylated cetuximab. Intensity of FITC and PE are shown in the cytogram, and fluorescence of FITC was readily detected in all four CRC cell lines (Fig. 2A). This result suggests that these CRC cell lines express EGFR, to which cetuximab binds on the cell surface). As indicated by the data shown in Figure. 2B, WiDR and SW480 cell lines expressed the highest levels of EGFR.

### Establishment of Cell Lines that Differentially Express EGFR

**Figure 3:**
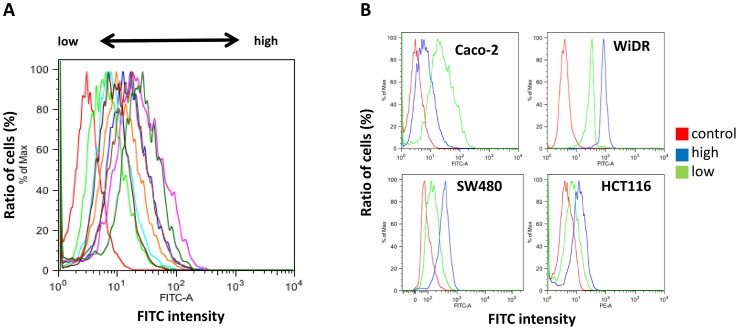
EGFR expression in subcloned CRC cell lines. (A) Histogram of WiDR subcloned cell lines showing differences in EGFR expression. Various EGFR expressions were seen in subcloned cell lines. (B) High and low EGFR subclones with high or low EGFR expression were selected on the basis of FCM results. Subclones derived from four CRC cell lines were analyzed, and the subclones exhibiting highest (blue) and lowest (green) levels of EGFR expression were selected for further studies. Controls are described in red line.

EGFR may be one of the most important genes for determining the phenotype of the cells. Therefore, the method to obtain various cell lines that had the least impact on the genetic background and showed a variety of EGFR expression was desirable. Limiting-dilution subcloning method is the major technique to derive different types of cells with the same genetic background [25]. We were able to isolate subclones of the CRC cell lines using the limiting dilution technique and determined the EGFR expression levels in each. In Figure 3A, the result of FACS with WiDR subcloned cells as well as various EGFR expressions are shown. We then selected cells from the subcloned cell lines that expressed the highest and lowest levels of EGFR (Fig. 3B). The mutation status of *KRAS*, *BRAF*, *PIK3CA*, and EGFR S492 ectodomain did not change in each subcloned cell lines.

### Sensitivity of CRC Cell Lines to Cetuximab

**Figure 4:**
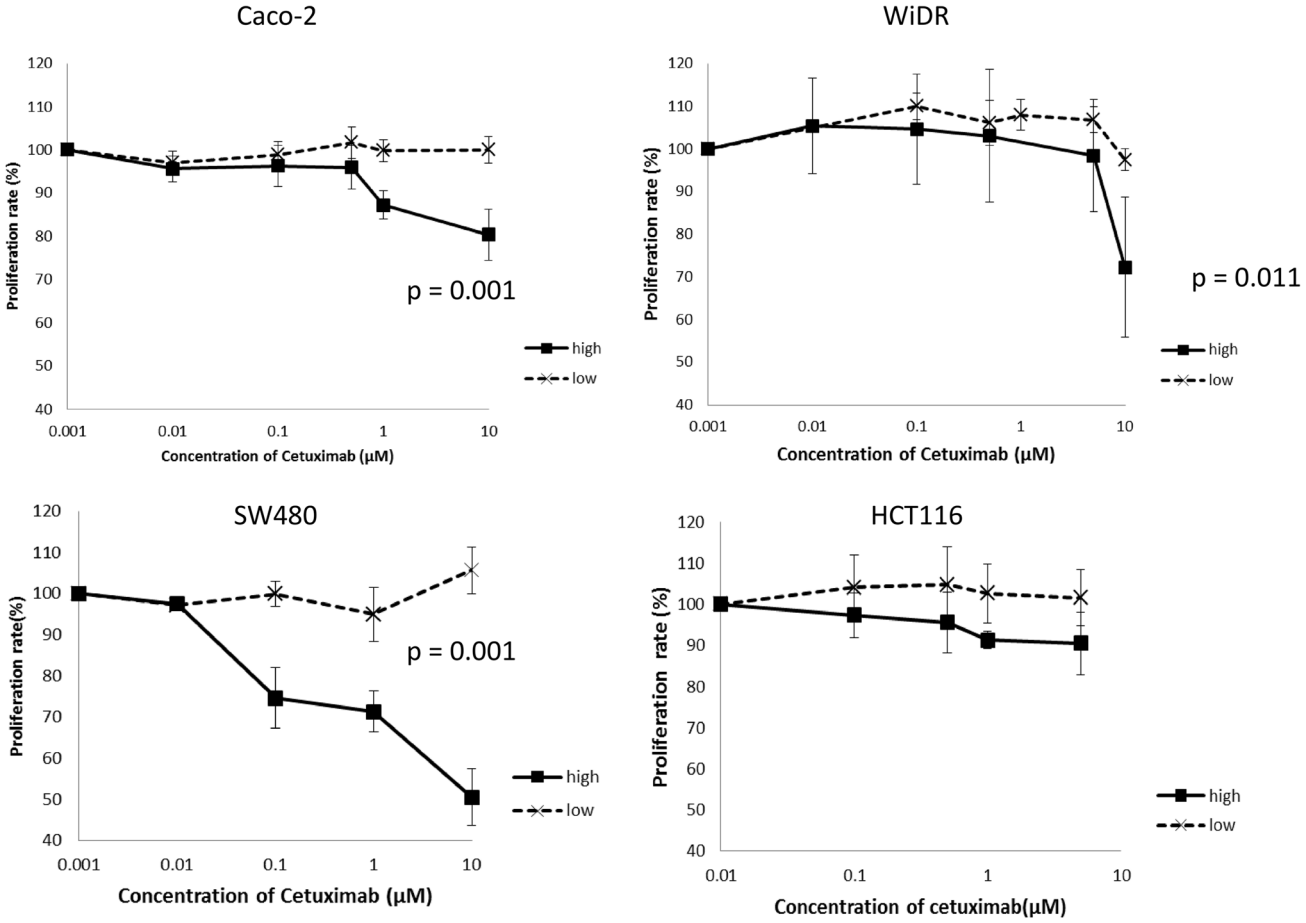
Proliferation assay of subcloned CRC cell lines. When the cetuximab was administered to low CRC subclones with low EGFR expression, weak inhibition of cell growth was observed. However, growth of subclones with high EGFR was strongly inhibited in comparison.

We conducted assays to determine whether cetuximab inhibits the proliferation of CRC cell lines. The growth of Caco-2 and SW480 were strongly inhibited, whereas intermediate inhibition of WiDR and no inhibition of HCT116 cell lines at the maximum dose of cetuximab were observed. To assess whether the EGFR expression level correlated with the proliferation inhibition effects of cetuximab, we used subcloned cell lines with high or low EGFR expression levels. To inhibit proliferation, subclones of Caco-2, SW480, and WiDR, which expressed the highest levels of EGFR, were most susceptible to effects of cetuximab (Fig. 4). However, response to cetuximab was not observed in subclones of these cell lines that had low EGFR expression levels. Subclones of HCT116, which in mass culture showed minimal response to cetuximab, did not respond to cetuximab whether they had high or low EGFR expression levels.

### Sensitivity to Cetuximab in Xenografts of Subcloned CRC Cell Lines

**Figure 5:**
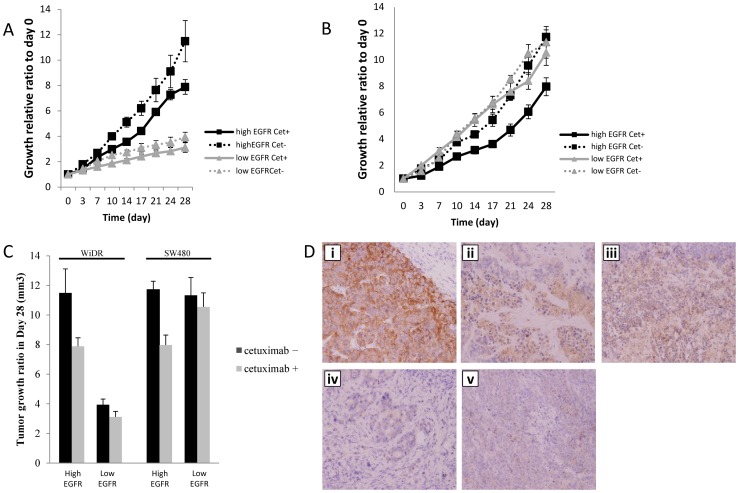
Susceptibility of tumors derived from xenografts to cetuximab. (A) Xenografts derived from WiDR subclone cells treated with cetuximab. Strong growth inhibition was seen in tumors derived from subclones with high EGFR expression compared with untreated mice; however, only slight inhibition of tumor growth induced by xenografts of subclones with low EGFR expression was observed. (B) Cetuximab treatment of tumors derived from xenografts of SW480 subclones cells. Similar results were also observed in tumors derived from SW480 xenografts i.e., strong growth inhibition of tumors derived from subclones with high EGFR expression compared with subclones with low EGFR expression. (C) Comparison of the tumor size on day 28. Tumors derived from subclones with high EGFR expression were significantly smaller in mice treated with cetuximab compared with tumors derived from subclones with low EGFR expression. (D) Representative immunohistochemical staining of 3+ EGF-R expression (i; A431), 2+ EGF-R expression (ii; WiDR High and iii; SW480 High) and 1+ EGF-R expression. (iv; WiDR Low and v; SW480 Low) in subcutaneous transplant tumor of the colon cancer cell lines.

Each of the subcloned cell lines derived from WiDR and SW480 were subcutaneously inoculated into nude mice (cetuximab, 30 mg/kg weekly for 4 weeks), and the difference of sensitivity to cetuximab was assessed *in vivo*. Cetuximab inhibited the growth of WiDR cells that expressed high levels of EGFR by 35% compared with untreated mice. In contrast, cetuximab treatment of a WiDR subclone that expressed low levels of EGFR was inhibited by 20% compared with untreated mice (Fig. 5A).

Xenografts derived from SW480 subclones that expressed high levels of EGFR were inhibited by 35% compared with untreated mice. However, xenografts derived from SW480 sublcones that expressed low levels of EGFR were inhibited by only 7% compared with untreated mice (Fig. 5B).

For tumors induced by subclones derived from WiDR or SW480 cultures, the tumor volume of groups treated with cetuximab was compared with that of the untreated groups after 28 days (Fig. 5C). Tumor volume was significantly lesser in the cetuximab-treated groups that were engrafted with cells expressing high levels of EGFR. In contrast, cetuximab treatment did not cause any significant difference in tumor volumes induced by subclones that expressed low levels of EGFR.

Based on the immunohistochemical staining, subcutaneous transplantation tumor of A431, positive control, showed 3+ EGFR expressions, whereas high WiDR and SW480 had 2+ EGFR expressions. In contrast, low WiDR and SW480 tumors showed less EGF-R expression (Fig. 5D). These results indicated that the post-treatment tumors still express EGFR.

## Discussion

Cetuximab, a chimeric IgG1 antibody that targets EGFR, was introduced to treat mCRC. To identify patients who would benefit the most from this biological therapy, detection of *KRAS* mutations was proposed as the principal biomarker [11], [26], [27]. Oncogenic mutations of *KRAS* are observed in about 40% (20%–50%) of sporadic CRC [28]–[31]. Mutations cause constitutive activation of the Ras/Raf/MAPK signaling pathway, which is independent of EGFR activation by ligand binding [32]. Karapetis *et al*. reported significant improvement in patients with wild-type *KRAS* in response to treatment with cetuximab [12].

EGFR status evaluated using immunohistochemical staining is considered another biomarker for the efficacy of anti-EGFR therapies. Thus, EGFR expression level was expected to correlate with the sensitivity and efficacy of cetuximab. Despite this hypothesis, results from a number of studies suggest that EGFR status and its expression level determined by immunohistochemical staining may not correspond to the efficacy of anti-EGFR therapy [8], [16]–[18]. Thus, a response to cetuximab was detected in patients with undetectable tumor-specific EGFR expression, leading to the conclusion that the response to cetuximab is independent of EGFR expression in tumor tissue [18].

The identification of additional genetic determinants of primary resistance to EGFR-targeted therapies in CRC is clearly a priority. The results presented in this study suggest that EGFR expression level, which is detected by cetuximab correlates with the efficacy of cetuximab treatment. Further, the assessment of EGFR expression using biotinylated cetuximab method may predict the clinical benefit of cetuximab treatment. Here we have demonstrated that proliferation of CRC cell lines was highly inhibited in cells that expressed EGFR at high levels, which suggests that EGFR expression levels detected by cetuximab correlate with the sensitivity of cells to cetuximab treatment. The efficacy of cetuximab in a xenograft model was also examined by comparing xenografts of CRC cell lines subclones that expressed high or low levels of EGFR. Greater growth inhibition of tumors was seen in tumor induced by subclones with high EGFR expression compared with those with low EGFR expression. However, we are not aware of any reports indicating that detection of EGFR by cetuximab is associated with the sensitivity of CRC to cetuximab treatment. Although further studies will be required to define the association between EGFR expression and sensitivity of tumor cells to cetuximab, our present results suggest that our method for detecting EGFR expression with cetuximab may provide a new biomarker.

We selected four CRC cell lines, Caco-2, WiDR, SW480, and HCT116 for our present study. These cell lines have been used in many other previous studies to assess the efficacy of cetuximab treatment. We also investigated the presence of mutations in *KRAS*, *BRAF*, *PIK3CA*, and EGFR ectodomain (Table 1). A strong response to cetuximab was observed in the Caco-2 cells, which lacked detectable mutations in these three genes; however, a response was also detected in SW480 cells, which harbors a *KRAS* and PIK3CA mutation. First, regarding *KRAS* mutation, the efficacy of cetuximab is associated with longer overall and progression-free survival among patients with chemotherapy-refractory colorectal cancer with p.G13D-mutated tumors than with other KRAS-mutated tumors [23]. Second, regarding PIK3CA mutation, Ogino *et al.* reported that *PIK3CA* mutations were associated with shorter cancer specific survival in patients with KRAS wild-type tumors in a series of stage I–III colorectal cancers [21]. However, De Roock *et al.* reported for the first time that PIK3CA exon 20 mutations are associated with worse outcomes compared with wild types, whereas exon 9 mutation was found to have no effect [22]. From these previous reports, our results were consistent with those of another study showing that cetuximab was effective in SW480, which has KRAS codon 13 mutation and PIK3CA exon 9 mutation, whereas no positive effect was seen in HCT116 with PIK3CA exon 20 mutation.

We also studied the WiDR and SW480 CRC cells to evaluate the efficacy of cetuximab efficacy using a mouse xenograft model. A proliferation assay demonstrated that a strong or mild response to cetuximab in mice engrafted with either WiDR or SW480. Caco-2, which showed strong response to cetuximab, was also used to establish xenograft model; however it failed to engraft the mice. The growth of tumors derived from the WiDR and SW480 xenografts was highly inhibited when subclones exhibited high EGFR expression compared to those with low EGFR expression. This suggests that the difference in quantity of EGFR correlates with cetuximab sensitivity. However, a limitation of this study is that there is a possibility of alterations in EGFR expression level during tumor progression. Although our new method to quantify EGFR expression using cetuximab requires further validation as a biomarker of the efficacy of anti-EGFR therapy, our results suggest that EGFR expression levels correlate with sensitivity of CRC to cetuximab.

The apparent discrepancies between the results of our present study and those previously reported can be explained as follows: First, EGFR is expressed in 40%–70% of colorectal cancers, and evidence suggests an association with survival [33]–[35]. Methods used to evaluate the quantity of EGFR in colorectal cancers include immunohistochemistry, ligand binding, immunoblotting, fluorescence in situ hybridization analysis and a variety of molecular techniques [36]. However, assessment of EGFR expression can be affected by immunoreactivity of normal tissues, differential EGFR reactivity of neoplasms from different areas of the bowel, and heterogeneity of reactivity within the colorectal carcinoma itself [17], [37]. Second, Scartozzi *et al.* reported that EGFR status in primary colorectal tumors differs from that of metastatic sites [38]. Third, Montagut *et al.* reported that EGFR S492R ectodomain mutation prevents cetuximab binding and confers resistance to cetuximab [19]. This finding suggests that EGFR quantification using immunohistochemistry staining detects either wild-type or mutated EGFR that do not bind cetuximab. Therefore, our new method may solve this problem because it depends on the ability of cetuximab to detect EGFR expression.

Many clinical trials have demonstrated the efficacy of adding cetuximab to treatment for mCRC [7], [8], [39]. Van Cutsem *et al*. performed a phase III study of first-line treatment, in which cetuximab was added to FOLFIRI and 5-FU-based multidrug chemotherapy with irinotecan [40]. The addition of cetuximab was associated with a significant increase in median progression-free survival compared with no cetuximab. Jonker *et al*., in another phase III study, compared anti-EGFR antibody therapy with best supportive care in patients [10]. In this study, cetuximab was added along with best supportive care and was associated with significant improvements compared with best supportive care alone. However, results from the majority of studies show a trend toward better survival if chemotherapy was combined with cetuximab but did not reveal a significant impact on CRC treatment.

The response rate of cetuximab treatment for CRC is 30%–40% [10]. Evidence suggests that *KRAS* mutations are the most effective predictors for selecting patients that will benefit from treatment; however, these mutations, by themselves, do not identify the best responders. Our present results suggests that this new method for detecting EGFR using biotinylated cetuximab may provide a means for detecting highly sensitive patients with wild-type *KRAS*. Although further investigation is required to assess our hypothesis, the technique described here may provide a more specific indication for implementing treatment of CRC with cetuximab.

In conclusion, results of the present study demonstrate that the EGFR expression levels, which were detected by cetuximab, may correlate with sensitivity to cetuximab-mediated inhibition of tumor cell growth. Therefore, we believe that these results may provide a new method to quantify EGFR expression, thereby enabling more effective selection of CRC patients that will benefit from cetuximab therapy.
